# The effect of prolonged spaceflight on cerebrospinal fluid and perivascular spaces of astronauts and cosmonauts

**DOI:** 10.1073/pnas.2120439119

**Published:** 2022-04-12

**Authors:** Giuseppe Barisano, Farshid Sepehrband, Heather R. Collins, Steven Jillings, Ben Jeurissen, James A. Taylor, Catho Schoenmaekers, Chloë De Laet, Ilya Rukavishnikov, Inna Nosikova, Liudmila Litvinova, Alena Rumshiskaya, Jitka Annen, Jan Sijbers, Steven Laureys, Angelique Van Ombergen, Victor Petrovichev, Valentin Sinitsyn, Ekaterina Pechenkova, Alexey Grishin, Peter zu Eulenburg, Meng Law, Stefan Sunaert, Paul M. Parizel, Elena Tomilovskaya, Donna R. Roberts, Floris L. Wuyts

**Affiliations:** ^a^Laboratory of Neuro Imaging, University of Southern California, Los Angeles, CA 90033;; ^b^Department of Radiology and Radiological Science, Medical University of South Carolina, Charleston, SC 29425;; ^c^Lab for Equilibrium Investigations and Aerospace, University of Antwerp, B-2610 Antwerp, Belgium;; ^d^Imec-Vision Lab, University of Antwerp, B-2610 Antwerp, Belgium;; ^e^Institute of Biomedical Problems, Russian Academy of Sciences, Moscow 123007, Russia;; ^f^Department of Radiology, National Medical Research Treatment and Rehabilitation Centre of the Ministry of Health of Russia, Moscow 125367, Russia;; ^g^Coma Science Group, University of Liège, 4000 Liège, Belgium;; ^h^Department of Radiology, Lomonosov Moscow State University, Moscow 119991, Russia;; ^i^Laboratory for Cognitive Research, HSE University, Moscow 101000, Russia;; ^j^Gagarin Cosmonauts Training Center, Star City 141160, Russia;; ^k^Institute for Neuroradiology, Ludwig-Maximilians-University Munich, 80539 Munich, Germany;; ^l^Department of Radiology, Alfred Health, Melbourne, VIC, 3181, Australia;; ^m^Department of Imaging and Pathology, Katholieke Universiteit Leuven, 3000 Leuven, Belgium;; ^n^Department of Radiology, Royal Perth Hospital, Perth, WA, 6000, Australia

**Keywords:** spaceflight, microgravity, perivascular space, brain, spaceflight-associated neuroocular syndrome

## Abstract

Long-duration spaceflight induces changes to the brain and cerebrospinal fluid compartments and visual acuity problems known as spaceflight-associated neuro-ocular syndrome (SANS). The clinical relevance of these changes and whether they equally affect crews of different space agencies remain unknown. We used MRI to analyze the alterations occurring in the perivascular spaces (PVS) in NASA and European Space Agency astronauts and Roscosmos cosmonauts after a 6-mo spaceflight on the International Space Station (ISS). We found increased volume of basal ganglia PVS and white matter PVS (WM-PVS) after spaceflight, which was more prominent in the NASA crew than the Roscosmos crew. Moreover, both crews demonstrated a similar degree of lateral ventricle enlargement and decreased subarachnoid space at the vertex, which was correlated with WM-PVS enlargement. As all crews experienced the same environment aboard the ISS, the differences in WM-PVS enlargement may have been due to, among other factors, differences in the use of countermeasures and high-resistive exercise regimes, which can influence brain fluid redistribution. Moreover, NASA astronauts who developed SANS had greater pre- and postflight WM-PVS volumes than those unaffected. These results provide evidence for a potential link between WM-PVS fluid and SANS.

A human mission to Mars and the building of a lunar outpost are two main goals several space agencies are aiming to achieve. This requires understanding how the human brain adapts to long-term exposure to reduced-gravity environments.

Widespread changes in brain structure and cerebrospinal fluid (CSF) redistribution were observed on postflight MRI in space flyers, including ventricular enlargement with no parenchymal atrophy (1–6), brain upward displacement with narrowing of the subarachnoid space at the vertex (VSA) (1, 3, 5), and alterations in water diffusivity (7). These changes correlate with spaceflight duration (8, 9), persist for several months after return to Earth (1–4), and suggest altered CSF homeostasis associated with spaceflight.

The clinical relevance of these alterations is unknown, but they might be related to spaceflight-associated neuro-ocular syndrome (SANS), a disorder characterized by ocular structural changes affecting ∼40 to 60% of NASA astronauts undergoing long-duration missions aboard the International Space Station (ISS) (10). While visual changes have been noted in Roscosmos (ROS) cosmonauts after spaceflight (1, 11, 12), there are no published reports characterizing them using the SANS classification developed by NASA.

Here, we aim to determine if spaceflight induces volumetric changes to the perivascular spaces (PVS), a brain-wide network of perivascular channels along which CSF–interstitial fluid (ISF) exchange occurs (13); to investigate the relationship between PVS dilation and spaceflight-associated alterations in VSA and lateral ventricles (LVs); and to analyze the relationship between these alterations and SANS. Furthermore, as differences in the adoption of microgravity countermeasures may influence the degree of the spaceflight-associated changes, we exploratively compared the alterations in these compartments for American, European, and Russian crews in a joint international study of pre- and postspaceflight brain MRI.

## Results

**Fig. 1.**
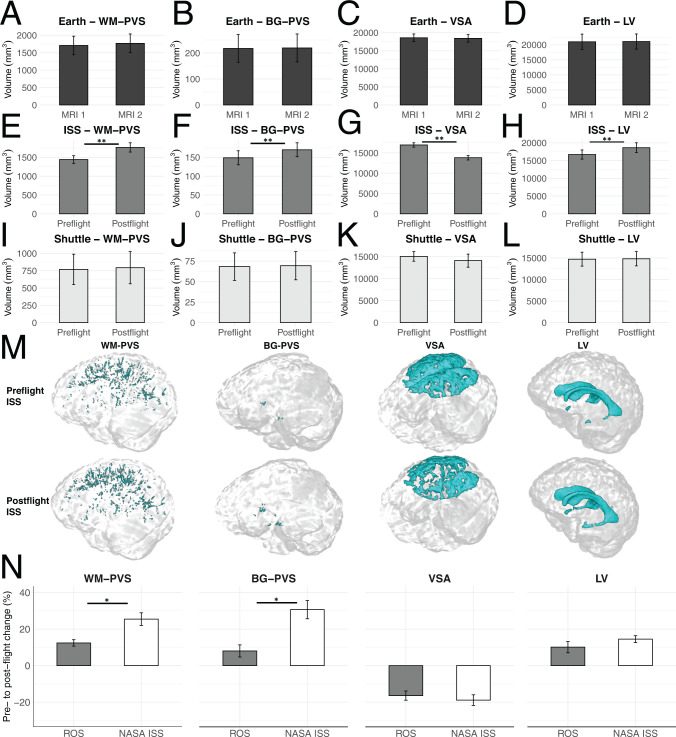
Controls on Earth do not show significant changes in PVS, VSA, or LV volumes after a 1-y follow-up (*A–D*). After long-duration spaceflight on the ISS, we observed a significant increase in PVS and LV and a decrease in VSA volumes (*E–H*). Short-duration spaceflight on the space shuttle was not associated with significant changes in PVS, VSA, or LV volumes (*I–L*). Examples of 3D masks (cyan) of WM-PVS, BG-PVS, VSA, and LV before and after long-duration spaceflight on the ISS (*M*). The postflight changes in PVS, but not in VSA and LV, were significantly higher in NASA astronauts than ROS cosmonauts (*N*). All data represent mean ± SEM. Paired (*A–L*) or independent samples *t* tests (*N*). *P ≤ 0.01; **P < 0.001.

We analyzed brain MRI scans acquired before and within 2 wk after long-duration spaceflight on the ISS (∼180 d) in 24 NASA astronauts (48.6 ± 5.4 y old), 13 ROS cosmonauts (47.4 ± 5.2 y old), and a small group of European Space Agency (ESA) astronauts (the number is not reported to protect the astronauts’ identity). Since on Earth, physiological changes occur over time in PVS, VSA, and LV, we also analyzed scans acquired with 1-y intervals in 13 age-matched healthy volunteers (46.2 ± 4.8 y old) who stayed on Earth; their white matter perivascular spaces (WM-PVS), basal ganglia perivascular spaces (BG-PVS), VSA, and LV volume changes between the first and second scans (Fig. 1 *A–D*) were not significant (5.3, 1.0, −0.9, and 0.7%, respectively). To correct for the time differences between the preflight scan and the launch day in all space flyers, these percentage changes were used to estimate the WM-PVS, BG-PVS, VSA, and LV volumes at launch day (indicated as “preflight” from now on) (*SI Appendix*).

The results of the statistical analysis are in Dataset S1.

No lesions were found in any of the scans. We observed significantly increased WM-PVS and BG-PVS volume, decreased VSA, and LV enlargement after long-duration spaceflight on the ISS (Fig. 1 *E–H* and *M*). No significant changes in these compartments occurred in seven NASA astronauts (46.1 ± 2.8 y old) who participated in 2-wk missions on the space shuttle (Fig. 1 *I–L*). The pre- to postflight WM-PVS increase was significantly correlated with VSA reduction; both WM-PVS and VSA changes were significantly correlated with mission duration. Age and preflight brain and WM-PVS volumes were not related to any of the postflight changes observed. Since experienced space flyers adapt differently to microgravity than first-time flyers (1), we also investigated the relationship between previous spaceflight experience and the PVS changes; BG-PVS, but not WM-PVS, changes were inversely correlated with previous spaceflight experience.

After long-duration spaceflight, the percentage increases in WM-PVS and BG-PVS volumes, but not VSA or LV volumes, were significantly higher in NASA astronauts than ROS cosmonauts (Fig. 1*N*). Since postflight scans in NASA astronauts were acquired closer to landing compared with postflight scans in ROS cosmonauts and the spaceflight-associated alterations in LV and VSA usually reverse months after return to Earth (1), we tested whether the changes in PVS were correlated with the interval between landing and the postflight scan; a significant inverse correlation was found for BG-PVS but not for WM-PVS, suggesting that the higher WM-PVS increase in NASA astronauts is not explained by a shorter landing–postflight MRI interval.

**Fig. 2.**
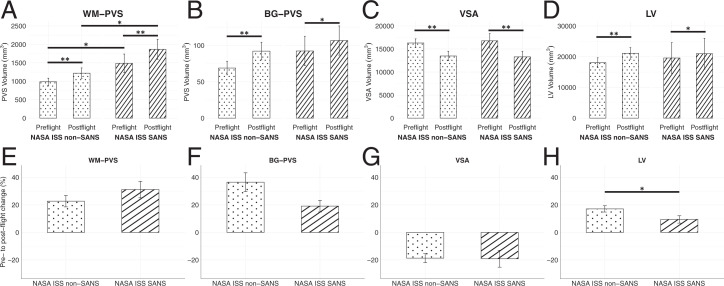
Preflight and postflight WM-PVS volumes (*A*) were significantly higher in NASA astronauts who developed SANS than those unaffected. A significant PVS (*A* and *B*) and LV (*D*) enlargement and VSA reduction (*C*) were observed in both groups (post hoc comparisons, mixed model ANOVA). The spaceflight-associated PVS dilation (*E* and *F*) and VSA reduction (*G*) were not significantly different between the groups, but a significantly greater LV enlargement (*H*) was observed in the non-SANS group (time by SANS interaction, mixed model ANOVA). Data represent mean ± SEM. *P < 0.05; **P ≤ 0.001.

Ophthalmologic records were available for the NASA astronauts who traveled on the ISS; eight (33.3%) developed clinical signs of SANS and presented greater pre- and postflight WM-PVS volumes than those unaffected, but similar pre- and postflight BG-PVS, VSA, and LV volumes (Fig. 2 *A–D*). The postflight percentage increases of PVS and reduction of VSA volumes were comparable in the two groups, but a significantly larger postflight increase in LV volume occurred in the non-SANS group (Fig. 2 *E–H*), as previously reported (9).

## Discussion

We showed that long-duration, but not short-duration, spaceflight is associated with PVS enlargement. The larger pre- and postflight WM-PVS volumes observed in NASA astronauts who developed SANS suggest that fluid accumulation in WM-PVS might play a pathophysiological role in SANS and that a higher WM-PVS volume at baseline might correspond to an increased risk of SANS. Higher body weight was found to be associated with an increased risk of SANS in astronauts (14); likewise, WM-PVS burden in healthy individuals is correlated with body mass index (15), which is also associated with higher intracranial pressure (ICP) (16). We speculate that the inverse correlation between WM-PVS and VSA changes could potentially indicate that the brain upward shift may contribute to WM-PVS dilation by obstructing major CSF–ISF efflux routes (e.g., arachnoid granulations, superior sagittal sinus, bridging veins), as previously observed (5). Space flyers’ sleep deprivation (17) and the elevated CO_2_ on the ISS (18) may also contribute to the PVS changes observed, as on Earth, PVSs are associated with poor sleep quality (19) and carbogen inhalation (95% O_2_/5% CO_2_) (20). On Earth, PVS enlargement is considered a nonspecific indicator of impaired brain health, being associated with several neurological conditions, including Alzheimer disease and small vessel disease (13). Our findings indicate that long-duration exposure to microgravity on the ISS may alter the CSF–ISF circulation in PVS, possibly impairing cerebral drainage systems like the paravascular/glymphatic pathway (21) and/or the intramural periarterial drainage pathway (22), and highlight the importance of a gravitationally maintained brain fluid homeostasis. PVSs were also identified in the optic nerve (23), and their dilation was speculated to be related to optic disk swelling in astronauts (24).

While LV expansion and VSA reduction were similar in ROS and NASA crews, the postflight WM-PVS enlargement was more prominent in NASA astronauts. Since age, mission duration, and environmental conditions were similar in NASA astronauts and ROS cosmonauts aboard the ISS, other factors must play a role in this difference. We hypothesize that differences in the adoption of microgravity countermeasures and/or exercise protocols may have influenced the extent of WM-PVS enlargement. For example, ROS cosmonauts undergo six lower body negative pressure (LBNP) sessions starting 2 wk prior to landing, while NASA and ESA astronauts do not typically do it. LBNP induces caudal displacement of fluids from the upper body by placing the legs and pelvis in a semiairtight chamber with negative pressure. An advanced resistive exercise device (ARED) is regularly used by space flyers to perform free weight exercises on the ISS, but the load and frequency of use are lower for ROS cosmonauts compared with NASA and ESA astronauts. Lifting heavy loads during resistive exercise is often accompanied by a brief Valsalva maneuver, inducing increased ICP and decreased cerebral blood flow and cerebrovascular transmural pressure (25), which can result in PVS fluid accumulation (13, 15). Although the effects of LBNP and ARED on the brain during spaceflight are unknown, they could partly explain the different WM-PVS changes detected in astronauts and cosmonauts. We cannot exclude that other factors (e.g., diet) might play a role in this difference. Further studies are required to confirm these hypotheses.

Our results reveal changes of WM-PVS following long-duration spaceflight, which differentially affect NASA and ROS crews and are linked to SANS, with implications for designing countermeasure strategies to support human health on future long-duration spaceflights and on Earth.

## Materials and Methods

The study comprising brain MRI data acquisition in ESA and ROS crews and controls was approved by the ESA Medical Board, the Committee of Biomedicine Ethics (Institute of Biomedical Problems, Russian Academy of Sciences), and the Human Research Multilateral Review Board. The study comprising brain MRI data review of NASA crews was approved by the institutional review boards at the NASA Johnson Space Center and the Medical University of South Carolina. All participants provided written informed consent.

Full methods are in *SI Appendix*. Briefly, three-dimensional (3D) high-resolution (1-mm^3^) T1-weighted images were acquired on 3-Tesla MRI machines. Preprocessing and LV segmentation were performed using FreeSurfer’s recon-all. PVSs were segmented via an automated quantification pipeline (26). VSA was segmented with Advanced Normalization Tools.

## Supplementary Material

Supplementary File

Supplementary File

## Data Availability

The NASA astronaut data are available upon application to the NASA Life Sciences Data Archive at the NASA Johnson Space Center (Houston, TX). Please contact D.R.R. (robertdr@musc.edu) for questions concerning NASA astronaut data, or F.L.W. (floris.wuyts@uantwerpen.be) and E.T. (Finegold@yandex.ru) for questions concerning ESA astronaut and ROS cosmonaut data. All other data are included in the manuscript and/or supporting information.
