# Supplementary material for: The effect of prolonged spaceflight on cerebrospinal fluid and perivascular spaces of astronauts and cosmonauts
Source: Proc Natl Acad Sci U S A. 2022 Apr 12;119(17):e2120439119. doi: 10.1073/pnas.2120439119 (PMC9169932; doi:10.1073/pnas.2120439119)
Supplement: Supplementary File [file pnas.2120439119.sapp.pdf]

PNAS
www.pnas.org

Supplementary Information for
The effect of prolonged Spaceflight on Cerebrospinal Fluid and Perivascular Spaces of Astronauts and Cosmonauts.

Giuseppe Barisano[1]*, Farshid Sepehrband[1], Heather R. Collins[2], Steven Jillings[3], Ben Jeurissen[4], Andrew J. Taylor[2], Catho Schoenmaekers[3], Chloë De Laet[3], Ilya Rukavishnikov[5], Inna Nosikova[5], Liudmila Litvinova[6], Alena Rumshiskaya[6], Jitka Annen[7], Jan Sijbers[4], Steven Laureys[7], Angelique Van Ombergen[3], Victor Petrovichev[6], Valentin Sinitsyn[8], Ekaterina Pechenkova[9], Alexey Grishin[10], Peter zu Eulenburg[11], Meng Law[12], Stefan Sunaert[13], Paul M. Parizel[14], Elena Tomilovskaya[5]†, Donna R. Roberts[2]*†, Floris L. Wuyts[3]*†

[1]Laboratory of Neuroimaging, University of Southern California; Los Angeles, USA
[2]Department of Radiology, Medical University of South Carolina; Charleston, USA
[3]Lab for Equilibrium Investigations and Aerospace, University of Antwerp; Antwerp, Belgium
[4]Imec-Vision Lab, University of Antwerp; Antwerp, Belgium
[5]Institute of Biomedical Problems, Russian Academy of Sciences; Moscow, Russia
[6]Therapeutic and Rehabilitation Center, Ministry of Health of Russia; Moscow, Russia
[7]Coma Science Group, University of Liège; Liège, Belgium
[8]Lomonosov Moscow State University; Moscow, Russia
[9]Laboratory for Cognitive Research, HSE University; Moscow, Russia
[10]Gagarin Cosmonauts Training Center; Star City, Russia
[11]Institute for Neuroradiology, Ludwig-Maximilians-University Munich; Munich, Germany
[12]Department of Radiology, Alfred Health; Melbourne, Australia
[13]Department of Imaging, KU Leuven; Leuven, Belgium
[14]Department of Radiology, Royal Perth Hospital; Perth, Australia

† These authors contributed equally to this work.

* Giuseppe Barisano, Donna R. Roberts, Floris L. Wuyts.

Email: giuseppe.barisano@loni.usc.edu (GB); robertdr@musc.edu (DRR); floris.wuyts@uantwerpen.be (FLW).

This PDF file includes:

Supplementary text
SI References

Other supplementary materials for this manuscript include the following:

Dataset S1

Supplementary Information Text

Extended methods

Experimental Design

This observational study includes brain magnetic resonance imaging (MRI) data that were collected per routine medical operations protocol in NASA astronauts, as well as from an ESA-endorsed prospective MRI study (“BRAIN-DTI”) in ROS cosmonauts, ESA astronauts, and control participants. Data collected in 24 NASA astronauts who participated in long-duration space missions to the ISS and in 7 NASA astronauts involved in missions of short duration in the Space Shuttle Program were provided to the investigators by the NASA Lifetime Surveillance of Astronaut Health Program. Brain MRI scans were acquired in 13 ROS cosmonauts and in a small group of ESA astronauts who participated in long-duration missions to the ISS. The number of ESA astronauts included in this study is not reported to maintain astronaut anonymity. The mean (SD) mission duration of all long-duration spaceflight subjects combined for the NASA, ROS, and ESA crews was 179.6 (47.8) days, while for the 7 NASA Shuttle astronauts was 14.7 (1.6) days. Brain MRI data were also available for 13 healthy control participants, who were of similar age and education level as the space crews. Demographic characteristics of the participants are reported in Dataset S1. The age was not significantly different between the five groups (P=.17, one-way ANOVA). Additionally, neither age nor brain volume were significantly different in the NASA astronauts who developed SANS (mean age 50.1 yo, median brain volume 1359.1 cm^3) compared with those in the non-SANS group (mean age 47.9 yo, median brain volume 1418.6 cm^3) (P=.35, independent samples t-tests and P=.15, Mann-Whitney U test, respectively; see also Dataset S1).

In space-flyers who underwent long-duration missions aboard the ISS, age was not correlated with WM-PVS changes (ρ=0.07, P=.65), BG-PVS changes (ρ=0.17, P=.29), VSA changes (ρ=0.14, P=.37), LV changes (ρ=-0.19, P=.23), mission duration (ρ=0.20, P=.21), previous spaceflight experience (ρ=0.13, P=.49) and interval landing-postflight MRI (ρ=-0.26, P=.09).

The study comprising brain MRI data acquisition in ESA and ROS crews and control subjects was approved by the ESA Medical Board, the Committee of Biomedicine Ethics of the Institute of Biomedical Problems of the Russian Academy of Sciences, and the Human Research Multilateral Review Board. The study comprising brain MRI data review of NASA crews was approved by the Institutional Review Boards at the NASA Johnson Space Center and the Medical University of South Carolina.

NASA astronauts and ROS cosmonauts were scanned a mean (SD) of 517.2 (148.7) and 121.0 (27.9) days before launch to the ISS (preflight MRI) and 2.6 (1.7) and 9.1 (3.2) days after return to Earth (postflight MRI), respectively. ESA astronauts were scanned 110.3 (55.8) days before launch and 5.8 (2.2) days after landing. NASA Shuttle astronauts were scanned 140.9 (58.7) days before launch and 14.4 (6.6) days after landing. Control participants were scanned twice, with a mean interval of 368.2 (101.1) days between scans.

All participants provided written informed consent to take part in this study.

Ophthalmologic data

Ophthalmologic records were available for the NASA ISS astronauts. We identified NASA astronauts who developed SANS based on the current diagnostic criteria for SANS, including optic disc edema, globe flattening, choroidal folds, hyperopic refractive error shifts and cotton wool spots (1, 2). While the definition of SANS is evolving and a new definition has recently been proposed based on incorporating changes in total retinal thickness (3), this measurement was not performed historically and, as such, was not available for all of the astronauts in our cohort. Therefore, for this study, we followed the current clinical diagnostic criteria for SANS classification.

MRI protocol

All brain scans were performed on 3T MRI machines equipped with 16-channel receiver head coils. The MRI protocol included the acquisition of 3D high-resolution T1-weighted structural images (voxel size 1×1×1 mm). NASA astronauts were scanned on a 3T Siemens Verio system (Siemens, Erlangen, Germany) in Houston, Texas, with the following parameters: T1-weighted

magnetization-prepared rapid acquisition of gradient-echo sequence; 176 sagittal slices; TR=2300ms; TE=2.98ms; TI=900ms; flip angle=7°. ROS cosmonauts and 9 controls were scanned on a GE Discovery MR750 3T MRI system (GE Healthcare, Milwaukee, Wisconsin) at the National Medical Research Treatment and Rehabilitation Center of the Ministry of Health of Russia in Moscow, Russia with the following parameters: T1-weighted fast spoiled gradient echo; 176 slices; TR=7.9ms; TE=3.06ms; TI=450ms; flip angle=12°.
The ESA astronauts and 4 controls were scanned on a 3T Siemens Biograph mMR system (Siemens, Erlangen, Germany) located at the German Aerospace Center (DLR) in Cologne, Germany, with the following parameters: T1-weighted magnetization-prepared rapid acquisition of gradient-echo sequence; 176 sagittal slices; TR=1900ms; TE=2.43ms; TI=900ms; flip angle=12°).

MRI data analysis
Preprocessing and parcellation of the T1-weighted images were performed using the *recon-all* module of *FreeSurfer* (v5.3.0, https://surfer.nmr.mgh.harvard.edu/) (4). Briefly, the following pre-processing steps were applied: motion correction, non-parametric non-uniform intensity normalization, Talairach transform computation, intensity normalization and skull stripping (5). Grey matter, white matter, and ventricular system parcellations were derived from the Desikan-Killiany atlas (6). The LV volume was computed by summing the left and right LV volumes obtained from the ventricular system parcellation in the native space.
BG-PVS and WM-PVS segmentation was performed on the parcellations in the native space previously derived from *FreeSurfer* via an automated quantification pipeline (7), as in previous studies (8–10). Briefly, an adaptive non-local mean filtering method (11) was applied on the T1-weighted images to remove bias intensity caused by the Rician noise in the MRI data. The PVS voxels were preserved by applying the filter only on high frequency spatial noise and by using a filtering patch with a radius of 1 voxel, which allows to preserve the signal intensities that are spatially repeated (11). The Frangi filter with the default, recommended parameters of α, β, and c (12) implemented in the *Quantitative Imaging Toolkit* (http://cabeen.io/qitwiki) (13) was subsequently applied to the denoised T1-weighted images for estimating the probability-like measure of “vesselness” in the native space (12). In order to maximize the PVS inclusion, we set the scale to a large range of 0.1–5 voxels. Finally, we applied a previously optimized threshold of 0.00002 (7, 9) to the vessel map in order to obtain a binary mask of PVS. The segmentation masks were reviewed blindly to the clinical and demographic information and manual corrections were applied with ITK-SNAP (version 3.8.0, www.itksnap.org) in case of mistakes in the segmentation.
Advanced Normalization Tools (ANTs v2.2.0, http://stnava.github.io/ANTs/) (14) was used to register all the T1-weighted images to a reference template, using a diffeomorphic image registration (15, 16), and to automatically segment the subarachnoid CSF space. From this segmentation mask, the fraction of the subarachnoid CSF space at the vertex (VSA) was obtained by selecting the first 30 slices (i.e., 30 mm) from the cranial vertex of the reference template towards the brain, as per the observations reported in previous publications (17–19). The volume of the VSA mask space was subsequently computed in the template space and used in statistical analyses.
The percentage change in WM-PVS, BG-PVS, LV, and VSA between the preflight and the postflight values was calculated as:

$$\%Change = \frac{Volume_{post} - Volume_{pre}}{Volume_{pre}} \times 100$$

Due to the different scanners adopted among groups, we used the percentage changes when comparing NASA with ROS MRI data, and absolute volumes when comparing intra-individual differences from the first to the second timepoint and when comparing groups of individuals scanned with the same machine (i.e., NASA ISS astronauts who developed SANS compared with those who did not develop SANS). All the segmentation software used here showed consistent and robust performance for T1-weighted images acquired on Siemens and GE scanners (9, 20–22).

Statistical analysis
Data were tested for normality using Shapiro-Wilk test. Pre- to post-flight changes in WM-PVS, BG-PVS, VSA, and LV were evaluated for the control, ROS, and NASA Shuttle groups with paired *t*-tests, and with mixed model ANOVAs for the NASA ISS group to identify changes separately for subjects with and without SANS. Group differences between ROS and NASA ISS were evaluated with independent samples *t*-tests. Due to the small sample size, ESA astronaut data were not included in this analysis and were characterized with descriptive rather than inferential statistics. To account for physiological brain changes over time on Earth and to accommodate the variability in time between the preflight MRI scan and launch, preflight values were adjusted to represent values on launch day based on the mean annual percentage change observed in our control group for WM-PVS, BG-PVS, VSA, and LV:

$$x_{adj} = x + (x \times y \times a)$$

where *x* is the value measured at the preflight scan, *y* is the interval between the preflight MRI scan and the launch in years, and *a* is the mean annual percentage change of the variable of interest (WM-PVS, BG-PVS, VSA, or LV) measured in the controls.
Spearman's correlation coefficients measured associations between changes in WM-PVS, BG-PVS, VSA, LV, spaceflight experience, mission duration and interval landing-postflight MRI.
A regression analysis was performed to test whether the WM-PVS volume measured at the postflight MRI scan was related to the baseline brain volume or age. Additional correlation analyses were performed to test the association of postflight changes in WM-PVS, VSA, and LV with age and baseline brain and WM-PVS volumes, as well as the association of preflight WM-PVS, VSA, and LV volumes with age and baseline brain volume.
Statistical significance was set at $\alpha<0.05$, two-tailed *P* values are reported, and post-hoc comparisons were conducted using the Šídák method (23) to correct for multiple comparisons. Analyses were conducted with SPSS (v25, IBM: Armonk, NY).

Limitations
There are several limitations in our study. One is the varying interval between the preflight scan and the launch across the astronauts and cosmonauts, since physiological changes occur in the brain with ageing. To reduce this limitation, we used the annual change rate detected in controls to compute in astronauts and cosmonauts the values expected on launch day based on their corresponding values measured at the preflight scan.
Another limitation is the lack of information about certain lifestyle factors (e.g., alcohol, smoking, and diet) which could have influenced the changes in PVS, in addition to ageing. Nevertheless, we believe that alcohol and smoking could not have played a significant role on the changes in PVS that we found after long-duration spaceflight on the ISS, since they are strictly prohibited on the ISS, while the control subjects on Earth could have access to them and still showed no significant changes in PVS at the second MRI scan. We also expect the intake of alcohol/tobacco to be minimal or absent in astronauts and cosmonauts during their leisure time on Earth, as one of the selection criteria for astronauts and cosmonauts requires them to be free from any dependency on drugs, alcohol, or tobacco. Additionally, according to our recent study in healthy adults, neither alcohol intake nor smoking are significantly associated with the PVS burden as quantified with our algorithm (8).
The varying interval between return to Earth and the postflight MRI scan is also a limitation. However, there was no correlation between WM-PVS changes and the interval between landing and the postflight MRI scan, which suggests that the higher WM-PVS changes measured in NASA ISS astronauts are not explained by the shorter landing-postflight MRI interval. Finally, the small number of ESA astronauts is also a limitation, which precluded the possibility to perform inferential statistics on this group.
Despite these limitations, this is the largest spaceflight MRI study to date and the first one analyzing PVS after spaceflight and comparing multiple intracranial CSF compartments in crews from different space agencies via the same computational tools by the same group of investigators.

SI References

1. A. G. Lee, *et al.*, Neuro-ophthalmology of space flight. *J. Neuro-Ophthalmology* **36**, 85–91 (2016).
2. A. G. Lee, *et al.*, Spaceflight associated neuro-ocular syndrome (SANS) and the neuro-ophthalmologic effects of microgravity: a review and an update. *npj Microgravity* **6** (2020).
3. K. Marshall-Goebel, *et al.*, Association of Structural Changes in the Brain and Retina After Long-Duration Spaceflight. *JAMA Ophthalmol.* (2021) https:/doi.org/10.1001/jamaophthalmol.2021.1400 (May 25, 2021).
4. B. Fischl, FreeSurfer. *Neuroimage* **62**, 774–781 (2012).
5. M. Reuter, N. J. Schmansky, H. D. Rosas, B. Fischl, Within-Subject Template Estimation for Unbiased Longitudinal Image Analysis. *Neuroimage* **61**, 1402–1418 (2012).
6. R. S. Desikan, *et al.*, An automated labeling system for subdividing the human cerebral cortex on MRI scans into gyral based regions of interest. *Neuroimage* **31**, 968–980 (2006).
7. F. Sepehrband, *et al.*, Image processing approaches to enhance perivascular space visibility and quantification using MRI. *Sci. Rep.* **9**, 12351 (2019).
8. G. Barisano, N. Sheikh-Bahaei, M. Law, A. W. Toga, F. Sepehrband, Body mass index, time of day and genetics affect perivascular spaces in the white matter. *J. Cereb. Blood Flow Metab.* **41**, 1563–1578 (2021).
9. F. Sepehrband, *et al.*, Volumetric distribution of perivascular space in relation to mild cognitive impairment. *Neurobiol. Aging* **99**, 28–43 (2021).
10. E. K. Donahue, *et al.*, Global and Regional Changes in Perivascular Space in Idiopathic and Familial Parkinson's Disease. *Mov. Disord.*, mds.28473 (2021).
11. J. V. Manjón, P. Coupé, L. Martí-Bonmatí, D. L. Collins, M. Robles, Adaptive non-local means denoising of MR images with spatially varying noise levels. *J. Magn. Reson. Imaging* **31**, 192–203 (2010).
12. A. F. Frangi, W. J. Niessen, K. L. Vincken, M. A. Viergever, "Multiscale vessel enhancement filtering" in *Medical Image Computing and Computer-Assisted Intervention— Miccai'98. 1496,* (Springer, Berlin, Heidelberg, 1998), pp. 130–137.
13. R. P. Cabeen, D. H. Laidlaw, A. W. Toga, Quantitative Imaging Toolkit: Software for Interactive 3D Visualization, Data Exploration, and Computational Analysis of Neuroimaging Datasets. in *Proceedings of the Joint Annual Meeting ISMRM-ESMRMB*, p. 8882.
14. B. B. Avants, N. Tustison, G. Song, Advanced Normalization Tools (ANTS). *Insight J.*, 1–35 (2009).
15. B. B. Avants, *et al.*, A reproducible evaluation of ANTs similarity metric performance in brain image registration. *Neuroimage* **54**, 2033–2044 (2011).
16. N. J. Tustison, B. B. Avants, Explicit B-spline regularization in diffeomorphic image registration. *Front. Neuroinform.* **7**, 39 (2013).
17. D. R. Roberts, *et al.*, Effects of Spaceflight on Astronaut Brain Structure as Indicated on MRI. *N. Engl. J. Med.* **377**, 1746–1753 (2017).
18. A. Van Ombergen, *et al.*, Brain Tissue–Volume Changes in Cosmonauts. *N. Engl. J. Med.* **379**, 1678–1680 (2018).
19. S. Jillings, *et al.*, Macro- and microstructural changes in cosmonauts' brains after long-duration spaceflight. *Sci. Adv.* **6**, eaaz9488 (2020).
20. N. J. Tustison, *et al.*, Large-scale evaluation of ANTs and FreeSurfer cortical thickness measurements. *Neuroimage* **99**, 166–179 (2014).
21. X. Han, *et al.*, Reliability of MRI-derived measurements of human cerebral cortical thickness: The effects of field strength, scanner upgrade and manufacturer. *Neuroimage* **32**, 180–194 (2006).
22. J. Jovicich, *et al.*, Brain morphometry reproducibility in multi-center 3T MRI studies: A comparison of cross-sectional and longitudinal segmentations. *Neuroimage* **83**, 472–484 (2013).
23. Z. Sidak, Rectangular Confidence Regions for the Means of Multivariate Normal Distributions. *J. Am. Stat. Assoc.* **62**, 626 (1967).
